# MiR-155 is involved in major depression disorder and antidepressant treatment via targeting SIRT1

**DOI:** 10.1042/BSR20181139

**Published:** 2018-12-21

**Authors:** Xun Wang, Bing Wang, Jianping Zhao, Caixing Liu, Xianpeng Qu, Yuhuan Li

**Affiliations:** 1 Department of Psychology, Qingdao Mental Health Center, Qingdao City, Shandong Province 266000, P.R. China; 2 Department of Pharmacy, Qingdao Women and Children’s Hospital, Qingdao City, Shandong Province 266000, P.R. China; 3 Department of Internal Medicine, Qingdao Eighth People’s Hospital, Qingdao City, Shandong Province 266000, P.R. China

**Keywords:** antidepressant treatments, depression, miR-155, SIRT1

## Abstract

Major depressive disorder (MDD) is a common mood disorder, and the treatment of MDD requires a variety of biopsychosocial approaches. The role of Silent information regulator 1 (SIRT1) in the regulation of MDD has recently been implicated. Here, we aimed to explore and elucidate the therapeutic effects of a microRNA, miR-155, in the treatment of MDD. With quantitative real-time PCR (qRT-PCR) analysis, we confirmed that cellular and serum levels of miR-155 were up-regulated in individuals with depression compared with those in healthy controls. TargetScan analysis indicated that SIRT1 is a target of miR-155, which was confirmed by dual-luciferase assay, qRT-PCR and Western blot analyses. Treatment of human neural progenitor cells with the antidepressant drug citalopram down-regulated miR-155 expression and up-regulated SIRT1 expression. These results suggest that miR-155 is an important factor in the pathophysiology of depression. miR-155 is a potential target for the development of new antidepressant treatments.

## Introduction

Major depression disorder (MDD) is a mood disorder that affects almost 350 million people worldwide. Evidenced by the rising suicide rates caused by depression, MDD is increasingly becoming a threatening public health problem [1]. MDD is symptomized by the lack of interest and pleasure in daily activities, worthless feeling and recurrent suicidal thoughts [2]. Over the last decades, neurobiological underpinnings of MDD have been the subjects of investigation [3], but the cellular and molecular mechanisms of MDD have yet to be understood.

Silent information regulator 1 (SIRT1) is a member of Sirtuin family, which is a nicotinamide–adenine dinucleotide-dependent deacylase, with broad regulatory functions in cell apoptosis, differentiation, autophagy, development, cancer metabolism and circadian rhythms [4]. SIRT1 was first identified in Han Chinese women as a MDD-related gene in a clinical trial conducted by CONVERGE (China, Oxford and Virginia Common-wealth University Experimental Research on Genetic Epidemiology) [5], whereby MDD patients were shown to possess a marked down-regulation of SIRT1 in blood samples. The dysregulated SIRT1 signaling plays an important role in depression-like behaviors [6,7]. Resveratrol, an antidepressant known to improve hyperanxiety status and attenuate depression-like behaviors [8], was demonstrated to activate SIRT1 [9]. However, the precise mechanism of SIRT1 regulation by antidepressants still remains unclear.

Recently, it has been widely recognized that protein-coding genes only comprise a small proportion of human genome and non-coding genes serve as important regulators of a variety of biological processes [10]. One of these non-coding genes is microRNAs (miRNAs), a class of short RNAs with the length of 17 to 22 nt that post-transcriptionally regulates gene expression by mediating target gene degradation. Abnormal miRNA expression is associated with a number of human diseases, including cancer, cardiovascular diseases etc [10]. The role of miRNAs in neural diseases, such as cerebral dysfunctions, schizophrenia, has also been recently explored [11]. There is a growing interest in clarifying the role miRNAs for psychiatric diseases, including MDD [12–14]. Thus far, a number of miRNAs have been identified to be associated with MDD, such as miR-132 and miR-124 [15]. However, despite the important role of SIRT1 in MDD, no link between the dysregulated miRNA expression and SIRT1 expression in MDD has been established.

Herein, we aim to investigate SIRT1-activating miRNAs associated with MDD. We hypothesized that miRNAs may synergize with SIRT1 to regulate MDD. In this report, we demonstrated that miR-155, among several miRNAs, was most prominently up-regulated in MDD patients. Previous evidence indicated that miR-155 mediates depression and anxiety-like behaviors in rodents [16], which drove us to select miR-155 as the subject of investigation in MDD. Our data suggested that miR-155 reciprocally interacted with SIRT1. The effects of common antidepressants, such as citalopram, amitriptyline and resveratrol, in regulating miR-155 and SIRT1 were also studied.

## Methods and materials

### Patients

All human studies were approved by Ethics Committee of Qingdao Mental Health Center. Written informed consent forms were acquired from patients. A group of MDD patients, who were admitted to the department of Psychiatry, Qingdao Mental Health Center, were selected as study subjects using the following inclusion criteria: major depression diagnosed in accordance to the ICD-10 or DSM IV criteria, with age between 18 and 35 years in Qingdao city. The severity of disease was assessed by the Hamilton Rating Scale for Depression (HAM-D, 17-item version). The characteristics of study subjects are summarized in Table 1. The exclusion criteria were as follows: current or previous treatment with antidepressants, mood stabilizers antipsychotics, or any regular treatment for a medical condition, or a personal history of primary substance abuse or primary organic disease.

**Table 1:** Clinical characteristics of MDD patients and healthy controls

Variable	MDD (*n*=68)	HC (*n*=42)	*P*-value
Gender (male/female)	47/21	25/17	0.312*
Age (mean ± SD)	27.68 ± 5.58	25.83 ± 5.09	0.5975^†[Table-fn T1TFN2]^
HAMD score, mean (SD)	22.47 ± 4.53	–	–

Abbreviations: HAM-D, Hamilton depression rating scale; HC, healthy control subjects; MDD, major depression disorder.

Notes:

* *P*-value for gender distribution was obtained by chi-square test.

† P-values were obtained by Student’s *t*-test.

### Cell culture and transfection

Human embryonic kidney cells (HEK 293) were cultured in DMEM medium supplemented with 10% fetal bovine serum (FBS), 100 U/ml penicillin and 100 μg/ml streptomycin (Invitrogen, U.S.A.) in an incubator maintained at 5% CO_2_ and 37°C. Human neural progenitor cells (NPCs) were maintained on culture plates coated with 5 mg/ml laminin (Sigma) and 200 μg/ml poly-l-ornithine hydrobromide (Sigma) and cultured in 70% DMEM (Invitrogen) supplemented with 30% Hams F12 (Mediatech), 1× penicillin–streptomycin (Invitrogen) and B-27 (Invitrogen). During expansion, media containing 20 ng/ml of human EGF (Sigma), 5 μg/ml heparin (Sigma) and FGF (R&D Systems) were used. To induce neural differentiation, cells were cultured in medium with growth factors until cells reached 90% confluence.

Lipofectamine 2000 (Invitrogen, U.S.A.) was used to transfect cells with pcDNA3.1-SIRT1 (TSINGKE, China), siRNA-SIRT1 (Cat.No: sc-40986, Santa Cruz, CA, U.S.A.), 50 nM miR-155 mimic (TSINGKE, China), 2′-O methylated single-stranded miR-155 antisense oligonucleotide (ASO-155, from TSINGKE, China) or RNA duplex control using following the instructions of the manufacture.

### SIRT1 agonist and antagonist treatment

NPCs were treated for 7 d with 50 μM resveratrol (Sigma), 1 μM EX527 (MCE) or a no-drug control (DMSO). All experiments were performed in triplicate.

### Citalopram and amitriptyline treatment

MTT assay was used to screen cytotoxic effects using human NPCs. Antidepressants were added at non-toxic concentrations. Cells were cultured continuously in either 50 μM citalopram hydrobromide, 5 μM amitriptyline hydrochloride or no-drug control for 24 h (acute) or 7 d (chronic). For patients, citalopram was used at 20 mg/day for 4 weeks, and then blood was collected in PAXgene blood RNA tubes (PreAnalytix, Switzerland). All experiments were performed in triplicate.

### Samples processing

Total RNA was extracted from blood samples or NPCs cells using the miRNeasy Mini Kit (Qiagen, Canada) according to manufacturer’s recommendations. The RNA was quantified using the Nanodrop 1000 (Thermo Scientific, U.S.A.).

### RT-PCR

cDNA library was created using the PrimeScript RT reagent Kit (Takara, China). PCR analysis was conducted on an Applied Biosystems 7500 Sequence Detection system (ABI, U.S.A.) using Roche FastStart Universal SYBR Green Master (Rox). The primers were synthesized by RiboBio (China). The levels of miR-155 and SIRT1 were normalized using U6 and GAPDH expression level as internal controls using the 2^−ΔΔ*C*^_t_ method. Fold changes relative to control samples were determined. The primer sequences used in the present study are included in Table 2.

**Table 2:** Primer sequences for RT-PCR

Gene	Primer sequences
miR-155	F: 5′-CGGCGGTTTAATGCTAATCGTGAT-3′;
	R: 5′-CCAGTGCAGGGTCCGAGGTAT-3′
SIRT1	F: 5′-TCAGTGTCATGGTTCCTTTGC-3′
	R: 5′-AATCTGCTCCTTTGCCACTCT-3′
U6	F: 5′-CGGCGGTCGTGAAGCGTTCCAT-3′
	R: 5′-CCAGTGCAGGGTCCGAGGTAT-3′
GAPDH	F: 5′-CACCATCTTCCAGGAGCGA-3′
	R: 5′-TCAGCAGAGGGGGCAGAGA-3′

### Western blot

Anti-SIRT1 antibody (1:2000, cat. no. ab32441) and anti-GAPDH antibody (1:2000, cat. no. ab8245) were acquired from Abcam. Cells were lysed and proteins were extracted with SDS lysis buffer (Beyotime, China), and resolved by SDS-PAGE. Following this, proteins were transferred to polyvinylidene difluoride (PVDF; Roche). Primary antibodies were applied to the membrane and incubated at 4°C overnight, followed by incubation with horseradish peroxidase-conjugated secondary antibodies and detection by the ECL method.

### Identification of miR-155 target

Gene targets of miR-155 were predicted using four miRNA target prediction databases: microRNA.org, RNA22, RNA Hybrid and TargetScan. Only the top eight genes predicted to target SIRT1 by all four databases were chosen for further validation.

### Statistical analyses

All numerical data are expressed as the mean ± S.D. Statistical differences among two groups were analyzed by Student’s *t*-test. Comparisons among multiple groups were made using one-way ANOVA followed by the Bonferroni post hoc test. Statistical significance was calculated using SPSS package version 17.0. *P*<0.05 was considered statistically significant.

### Availability of data and materials

The analyzed data sets generated during the study are available from the corresponding author on reasonable request.

## Results

### MiR-155 targets SIRT1 in MDD patients

**Figure 1:**
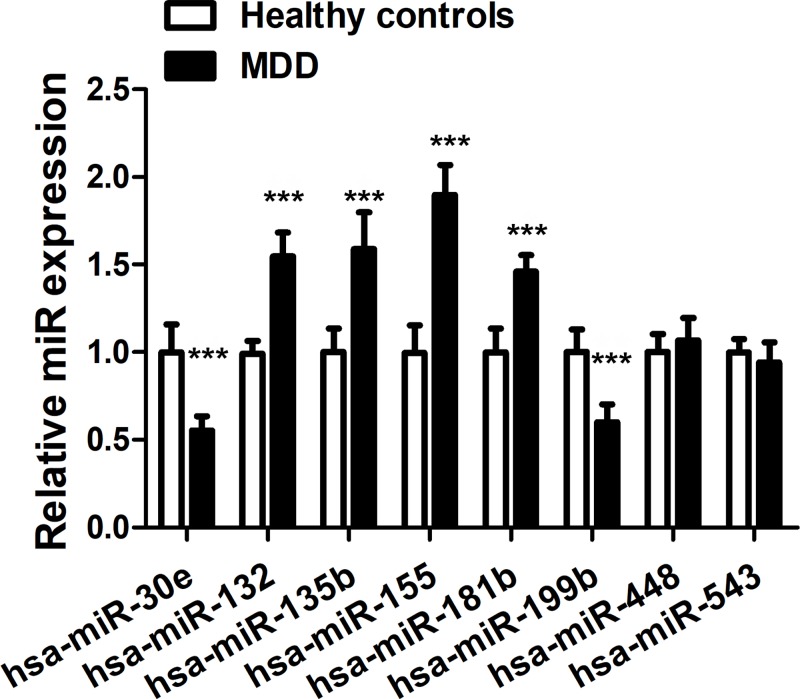
Expression of miRNAs potentially targeting SIRT1 RT-PCR was performed to detect the expression of 8 miRNAs in the 20 blood samples of MDD patients and 20 healthy controls, the results indicated that miR-155 was down-regulated the most; ***P<0.001.

With the aim to screen for a SIRT1-targeting miRNA associated with MDD, we compared the expression of eight candidate miRNAs, including hsa-miR-30e, hsa-miR-132, hsa-miR-135b, hsa-miR-155b, hsa-miR-181b, hsa-miR-199b, hsa-miR-448 and hsa-miR-543, in the blood samples of healthy and MDD patients. As shown in Figure 1, six of the miRNAs demonstrated significant difference in healthy and MDD patients, among which has-miR-155 showed the most prominent up-regulation (*F* = 0.005, *t* = −9.551, d*f* = 9.949, *P*<0.001) in MDD patients. Based on this, we proceeded to focus on the role of miR-155 in MDD and its interaction with SIRT1.

**Figure 2:**
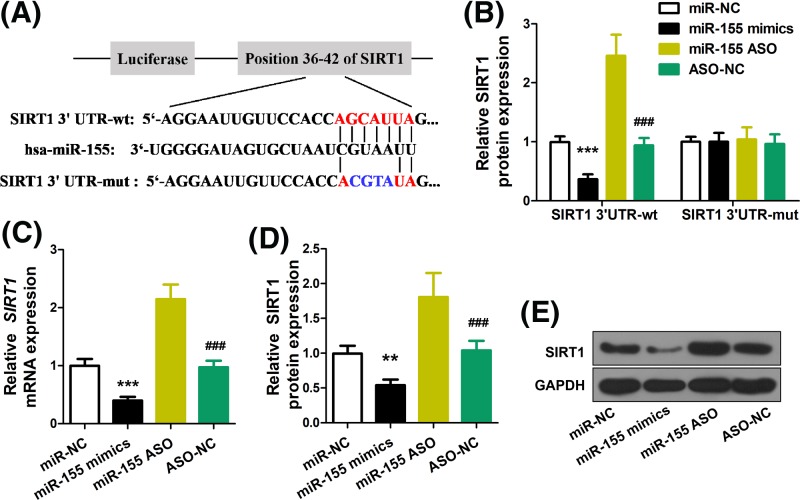
miR-155 targets SIRT1 to repress its expression (**A**) Scheme with bioinformatic analysis that showed miR-155 can target SIRT1. (**B**) HEK293 cells were transfected with miR-155 mimics or ASO to analyze the effect on the luciferase intensity of SIRT1-3′UTR reporter. (**C**) Real-time PCR was used to analyze the effect of miR-155 on SIRT1 mRNA. (**D** and **E**) Western blot was used to analyze the effect of miR-155 on SIRT1 protein level; **P<0.01, ***P<0.001 vs. miR-NC, ^###^*P*<0.001 vs. miR-155 ASO.

Next, we performed TargetScan analysis and found that a binding site exists on the 3’-UTR of SIRT1 (Figure 2A). The interaction between miR-155 and SIRT1 was further verified by luciferase assay, whereby HEK293 cells were transfected with miR-155 mimics, followed by monitoring activity of SIRT1 reporter. As shown in Figure 2B, while miR-155 mimics down-regulated the activity of wild-type SIRT1 3’-UTR reporter, no changes in the mutant SIRT1 3’-UTR reporter were induced by transfecting miR-155 mimics. On the contrary, transfecting miR-155 siRNA (ASO) increased the activity of wild-type SIRT1 reporter, but no changes were brought by transfecting non-coding ASO. Consistently, analyses of SIRT1 mRNA (Figure 2C) and protein (Figure 2D,E) showed that miR-155 overexpression reduced SIRT1 levels while miR-155 ASO increased SIRT1 expression. No changes were induced by transfecting miR-NC or ASO-NC. These evidence indicate that miR-155 suppresses the expression of SIRT1.

### Citalopram induces miR-155 down-regulation in neural progenitor cells

**Figure 3:**
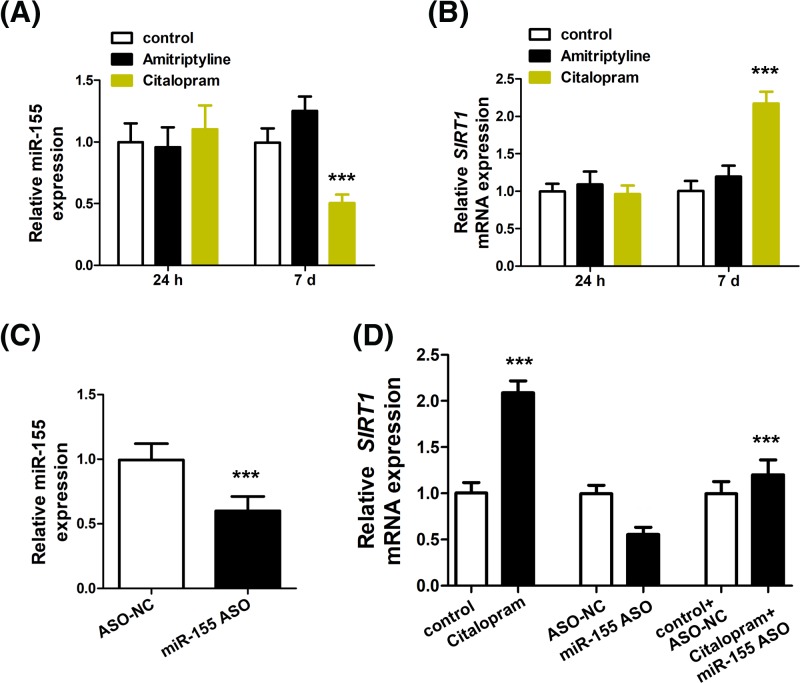
The alteration of miR-155 and SIRT1 after citalopram treatment RT-PCR was performed to detect the expression of miR-155 (**A**) and SIRT1 (**B**). (**C**) NPCs were transfected with ASO-NC or miR-155 ASO, and miR-155 was detected by RT-PCR at 7 d. (**D**) NPCs were treated with citalopram, miR-155 ASO, or co-treated with citalopram and miR-155 ASO, then the expression of SIRT1 was detected by RT-PCR at 7 d. ***P<0.001 vs. control or ASO-NC.

Citalopram is a common antidepressant and to further explore the link between miR-155 and MDD, we also investigated how the treatment of citalopram altered miR-155 levels. To this end, NPCs were incubated with citalopram. Amitriptyline treatment was used as a reference as amitriptyline does not affect SIRT1 expression [17]. Interestingly, citalopram efficiently down-regulated miR-155 level (Figure 3A) but up-regulated SIRT1 level (Figure 3B) at day 7. However, no significant alterations in miR-155 and SIRT1 levels were seen at 24 h. At day 7, NPC treated with miR-155 mimic demonstrated marked up-regulation of miR-155 (Figure 3C) compared to those treated with ASO-NC (*F* = 0.001, *t* = 5.738, d*f* = 9.881, *P*<0.001). In line with this, miR-155 mimic transfection also resulted in down-regulation of SIRT1 (Figure 3D). Treatment with both miR-155 mimic and citalopram, whereas, attenuated the change in SIRT1 (Figure 3D).

**Figure 4:**
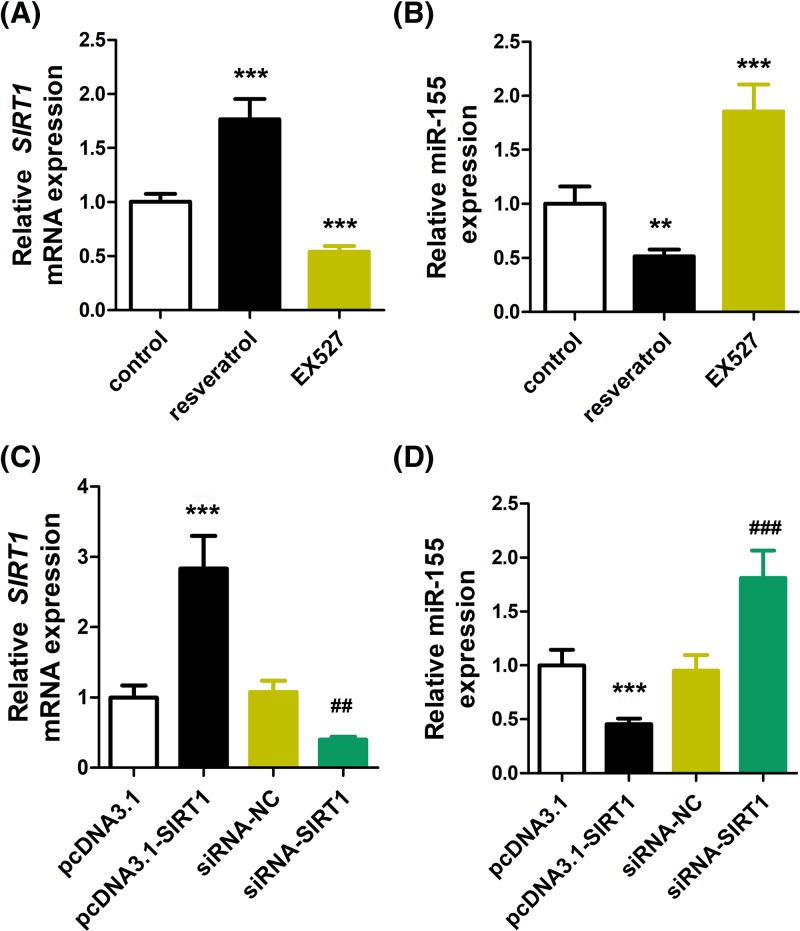
SIRT1 can regulate miR-155 NPCs were treated with the resveratrol and EX527 for 7 d. Then, the expression of SIRT1 (**A**) and miR-155 (**B**) were detected. (**C**) Transfection efficiency was detected with RT-PCR when the cells were transfected with pcDNA3.1-SIRT1 and siRNA-SIRT1. (**D**) The expression of miR-155 was detected by RT-PCR. **P<0.01, ***P<0.001 vs. control, ^##^*P*<0.01, ^###^*P*<0.001 vs. siRNA-NC.

To validate the interaction between SIRT1 and miR-155, the effects of other antidepressants, including resveratrol and EX527, were used to treat NPCs, followed by evaluating SIRT1 and miR-155 expression. As a result, SIRT1 was increased and decreased by resveratrol and EX627, respectively (Figure 4A). On the contrary, miR-155 levels were shown to be down-regulated and up-regulated by resveratrol and EX527, respectively (Figure 4B). These data agree with the notion that EX527 is an inhibitor of SIRT1, while resveratrol is an inducer [18–20].

### SIRT1 regulates miR-155 expression

Further, SIRT1 overexpression and silencing were induced by transfecting pcDNA3.1-SIRT1 and siRNA-SIRT1, respectively (Figure 4C). Impacts on miR-155 by gene transfection were similar to those by resveratrol and EX527 (Figure 4D). Together, these data suggested that SIRT1 can regulate miR-155 expression.

### Citalopram alleviates the miR-155 down-regulation and SIRT1 up-regulation in MDD patients

**Figure 5:**
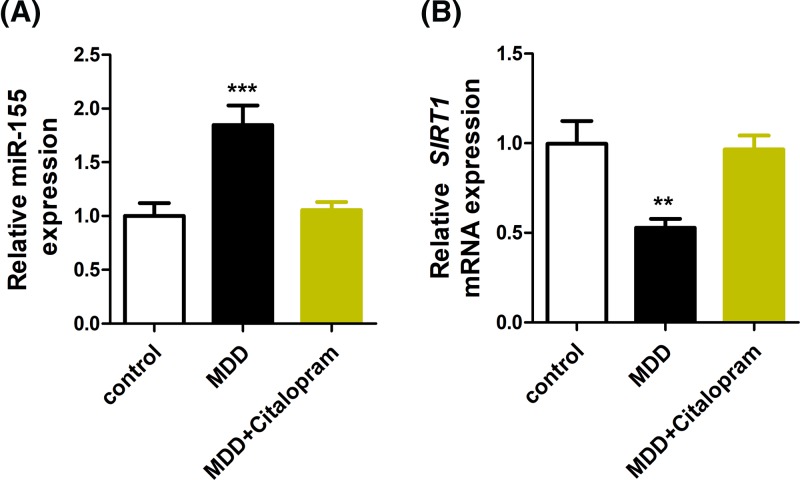
Effects of citalopram treatment on miR-155 and SIRT1 We investigated blood samples from 42 healthy control subjects and 68 MDD patients before and after citalopram treatment. The RT-PCR was used to detect the expression of miR-155 (**A**) and SIRT1 (**B**); **P<0.01, ***P<0.001.

Given the effects of citalopram in attenuating the alterations of miR-155 and SIRT1, we proceeded to test if MDD patients with citalopram treatment have normal miR-155 and SIRT1 expression. As shown in Figure 5, while MDD patients demonstrated significantly up-regulated miR-155 (Figure 5A) and down-regulated SIRT1 (Figure 5B) in blood samples, citalopram treatment brought these levels to normal. Therefore, miR-155 and SIRT1 levels clearly correlated to the therapeutic effects of citalopram.

## Discussion

Despite the recognition that miRNAs are important regulatory molecules in a large array of human malignancies, rare studies have been devoted to the elucidation of miRNAs in MDD regulation. The goal of the present study was to investigate the pathogenesis of MDD and explore potential miRNA markers of depression. With escalating MDD incidence rates, understanding of the molecular underpinnings of MDD is valuable for the development or enhancement of treatments for MDD patients. Here, we report that MDD patients are characterized by up-regulation of miR-155 in blood samples. These data suggest that miR-155 could potentially be used as a surrogate biomarker for anxiety and depression severity. Indeed, substantial efforts have been made recently to characterize plasma miRNAs or cerebrospinal fluid (CSF) miRNAs for monitoring treatment responses of antidepressants [14,21]. However, it is worth noting that in a prior study, miR-155 up-regulation was only observed in CSF, but not in serum, of MDD patients [13], which contradicts our findings. Our study only contains 20 samples from healthy and MDD patients, and therefore is limited by the small sample size. Further investigations of the correlation between miR-155 expression and MDD, with a larger sample size and comprehensive genetic background, are important to validate the serum up-regulation of miR-155 in MDD patients. Besides, we also demonstrated that miR-132 is up-regulated in MDD patients, in agreement to a previous study [15]. The other miRNAs, including hsa-miR-30e, hsa-miR-135b, hsa-miR-181b and hsa-miR-199b, which showed significant difference between healthy and MDD individuals, may also be the subject of further investigation to fully elucidate the molecular mechanisms of MDD.

The putative MDD gene, SIRT1, was demonstrated as a target of miR-155, in our luciferase assay, qRT-PCR and Western blot analyses. This finding is in agreement to a previous study on the interaction between miR-155 and SIRT1 in diabetic kidney disease [22]. miR-155 has been extensively studied as a regulatory molecule in cancer [23], inflammatory diseases [24] etc. miR-155 overexpression is often considered promoter of cancer malignancies [23,24]. The role of miR-155 is, however, complicated given the fact that miR-155 acts as suppressor of inflammatory diseases, such as atherosclerosis [25]. Therefore, caution has to be taken on clinical translation of miR-155-based treatments due to its conflicting role in various diseases. Moreover, SIRT1 is also a critical mediator in a large array of human diseases, such as cancer [26], inflammation [27], cardiovascular diseases [28] etc. Despite that SIRT1 activation is generally thought of an ameliorating factor in inflammatory diseases [27,28], conflicting evidence exist in regard to its role in cancer [26,29]. Notably, we show that the interaction between miR-155 and SIRT1 is reciprocal, i.e. overexpression and silencing of SIRT1 down-regulates and up-regulates miR-155 expression, respectively. This data suggested that there exists other mechanism in the regulation of miR-155 associated with SIRT1, which however we did not probe into.

Our study also showed the activation of SIRT1 by citalopram, similar to the effects of resveratrol in activating SIRT1 as demonstrated previously [18,19]. However, the other antidepressant, amitriptyline, showed no effects in SIRT1 regulation, suggesting that its regulatory effects in MDD are mediated through other mechanisms. As a control, EX527, an inhibitor of SIRT1 [20], exerted contradictory effects compared with citalopram. These evidence unveiled differential mechanisms of antidepressants in MDD. Given the interaction between miR-155 and SIRT1, we also showed that transfection of miR-155 mimics was also capable of suppressing SIRT1 expression, implicating the application of this gene therapy approach in MDD treatment. In the present study, our results are limited to *in vitro* evaluation of the effects of miR-155 in NPCs. Further *in vivo* study is needed to validate the applicability of this new therapy.

## Conclusions

In sum, we report that miR-155 is a biomarker molecule associated with MDD. SIRT1 is a target of miR-155, and miR-155 suppresses SIRT1 expression. Citalopram down-regulates miR-155 expression and up-regulates SIRT1 expression in NPCs. These data extend the understanding of MDD pathogenesis and potentiate a new treatment for MDD based on miR-155.

## Abbreviations

CSF: cerebrospinal fluid
MDD: major depressive disorder
NPC: neural progenitor cell
qRT-PCR: quantitative real-time PCR

## References

[1] Sobocki P. (2006) Cost of depression in Europe. J. Ment. Health Policy Econ. 9, 87–98 17007486

[2] Otte C. (2016) Major depressive disorder. Nat. Rev. Dis. Primers 2, 16065 10.1038/nrdp.2016.65 27629598

[3] Disner S.G. (2011) Neural mechanisms of the cognitive model of depression. Nat. Rev. Neurosci. 12, 467–477 10.1038/nrn3027 21731066

[4] Lu G. (2018) Role and possible mechanisms of Sirt1 in depression. Oxid. Med. Cell Longev. 2018, 8596903 10.1155/2018/8596903 29643977 PMC5831942

[5] consortium C. (2015) Sparse whole-genome sequencing identifies two loci for major depressive disorder. Nature 523, 588–591 10.1038/nature14659 26176920 PMC4522619

[6] Abe-Higuchi N. (2016) Hippocampal Sirtuin 1 signaling mediates depression-like behavior. Biol. Psychiatry 80, 815–826 10.1016/j.biopsych.2016.01.009 27016384

[7] Kishi T. (2010) SIRT1 gene is associated with major depressive disorder in the Japanese population. J. Affect. Disord. 126, 167–173 10.1016/j.jad.2010.04.003 20451257

[8] Ali S.H. (2015) Resveratrol ameliorates depressive-like behavior in repeated corticosterone-induced depression in mice. Steroids 101, 37–42 10.1016/j.steroids.2015.05.010 26048446

[9] Howitz K.T. (2003) Small molecule activators of sirtuins extend Saccharomyces cerevisiae lifespan. Nature 425, 191–196 10.1038/nature01960 12939617

[10] Piletic K. and Kunej T. (2016) MicroRNA epigenetic signatures in human disease. Arch. Toxicol. 90, 2405–2419 10.1007/s00204-016-1815-7 27557899

[11] Hoye M.L. (2017) MicroRNA profiling reveals marker of motor neuron disease in ALS models. J. Neurosci. 37, 5574–5586 10.1523/JNEUROSCI.3582-16.2017 28416596 PMC5452343

[12] O’Connor R.M. (2016) All roads lead to the miRNome: miRNAs have a central role in the molecular pathophysiology of psychiatric disorders. Trends Pharmacol. Sci. 37, 1029–1044 10.1016/j.tips.2016.10.004 27832923

[13] Wan Y. (2015) Identification of differential microRNAs in cerebrospinal fluid and serum of patients with major depressive disorder. PLoS One 10, e0121975 10.1371/journal.pone.0121975 25763923 PMC4357380

[14] Oved K. (2012) Genome-wide miRNA expression profiling of human lymphoblastoid cell lines identifies tentative SSRI antidepressant response biomarkers. Pharmacogenomics 13, 1129–1139 10.2217/pgs.12.93 22909203

[15] Fang Y. (2018) Changes in miRNA-132 and miR-124 levels in non-treated and citalopram-treated patients with depression. J. Affect. Disord. 227, 745–751 10.1016/j.jad.2017.11.090 29689690

[16] Arslan F. (2013) Mesenchymal stem cell-derived exosomes increase ATP levels, decrease oxidative stress and activate PI3K/Akt pathway to enhance myocardial viability and prevent adverse remodeling after myocardial ischemia/reperfusion injury. Stem Cell Res. 10, 301–312 10.1016/j.scr.2013.01.002 23399448

[17] YU H.-y. (2017) Baicalin treatment regulates hyperactivity of HPA axis and alters SIRT1 related inflammation in the hypothalamus in a model of depression. Chin. J. Pharmcol. Toxicol. 5, 054

[18] Ghosh S., Liu B. and Zhou Z. (2013) Resveratrol activates SIRT1 in a Lamin A-dependent manner. Cell Cycle 12, 872–876 10.4161/cc.24061 23439428 PMC3637344

[19] Borra M.T., Smith B.C. and Denu J.M. (2005) Mechanism of human SIRT1 activation by resveratrol. J. Biol. Chem. 280, 17187–17195 10.1074/jbc.M501250200 15749705

[20] Xu S. (2016) SIRT1/3 activation by resveratrol attenuates acute kidney injury in a septic rat model. Oxid. Med. Cell Longev. 2016, 7296092 10.1155/2016/7296092 28003866 PMC5149703

[21] Zhang Q.L. (2014) A preliminary analysis of association between plasma microRNA expression alteration and symptomatology improvement in Major Depressive Disorder (MDD) patients before and after antidepressant treatment. Eur. J. Psychiatry 28, 252–264 10.4321/S0213-61632014000400006

[22] Wang Y. (2018) Role of p53/miR-155-5p/sirt1 loop in renal tubular injury of diabetic kidney disease. J. Transl. Med. 16, 10.1186/s12967-018-1486-7 PMC5975703 29848325

[23] Habbe N. (2009) MicroRNA miR-155 is a biomarker of early pancreatic neoplasia. Cancer Biol. Ther. 8, 340–346 10.4161/cbt.8.4.7338 19106647 PMC2692997

[24] Tili E., Croce C.M. and Michaille J.J. (2009) miR-155: on the crosstalk between inflammation and cancer. Int. Rev. Immunol. 28, 264–284 10.1080/08830180903093796 19811312

[25] Li X.Y. (2016) miR-155 acts as an anti-inflammatory factor in atherosclerosis-associated foam cell formation by repressing calcium-regulated heat stable protein 1. Sci. Rep. 6, 10.1038/srep21789 PMC4761895 26899994

[26] Huffman D.M. (2007) SIRT1 is significantly elevated in mouse and human prostate cancer. Cancer Res. 67, 6612–6618 10.1158/0008-5472.CAN-07-0085 17638871

[27] Yoshizaki T. (2010) SIRT1 inhibits inflammatory pathways in macrophages and modulates insulin sensitivity. Am. J. Physiol.-Endocrinol. Metab. 298, E419–E428 10.1152/ajpendo.00417.2009 19996381 PMC2838524

[28] Chong Z.Z. (2012) Targeting cardiovascular disease with novel SIRT1 pathways. Fut. Cardiol. 8, 89–100 10.2217/fca.11.76 22185448 PMC3254055

[29] Firestein R. (2008) The SIRT1 deacetylase suppresses intestinal tumorigenesis and colon cancer growth. PLoS One 3, e2020 10.1371/journal.pone.0002020 18414679 PMC2289879

